# Oxidative stress and sperm function: A systematic review on evaluation and management

**DOI:** 10.1080/2090598X.2019.1599624

**Published:** 2019-04-24

**Authors:** Sulagna Dutta, Ahmad Majzoub, Ashok Agarwal

**Affiliations:** a Faculty of Dentistry, MAHSA University, Selangor, Malaysia; b Department of Urology, Hamad Medical Corporation, Doha, Qatar; c Department of Urology, Weill Cornell Medicine – Qatar, Doha, Qatar; d American Center for Reproductive Medicine, Cleveland Clinic, Cleveland, USA

**Keywords:** Male infertility, oxidative stress, reactive oxygen species, sperm function

## Abstract

**Objective**: To review and present the most distinct concepts on the association of reactive oxygen species (ROS) with male reproduction.

**Methods**: The Preferred Reporting Items for Systematic Reviews and Meta Analyses (PRISMA) guidelines were used to search PubMed, Medline, EMBASE, and the Cochrane electronic databases for studies investigating the role of oxidative stress (OS) on sperm function.

**Results**: The literature search yielded 1857 studies, of which 1791 articles were excluded because of irrelevance of data, non-English language, non-human nature or because they were case reports or commentaries. All included studies were reviews (46), meta-analyses (one), original research studies (18) and guideline articles (one). The studies were published between 1984 and 2018. Under normal physiological conditions, ROS are vital for sperm maturation, hyperactivation, capacitation, acrosome reaction, as well as fertilisation. However, a number of endogenous and exogenous causes may induce supra-physiological levels of ROS resulting in lipid peroxidation, sperm DNA fragmentation and apoptosis, and consequently infertility. Several laboratory testing methods can be used in infertile men to diagnose OS. Treatment usually involves antioxidant supplementation and, when possible, elimination of the causative factor.

**Conclusion**: OS is an important cause of male factor infertility. Its assessment provides essential information that can guide treatment strategies aimed at improving the male’s reproductive potential.

**Abbreviations:** bp: base-pair; CAT: catalase; LPO: lipid peroxidation; MDA: malondialdehyde; MiOXSYS: Male Infertility Oxidative System; mtDNA: mitochondrial DNA; NAD(PH): nicotinamide adenine dinucleotide (phosphate); NO: nitric oxide; 8-OHdG: 8-hydroxy-2’-deoxyguanosine; ORP: oxidation–reduction potential; OS: oxidative stress; PKA: protein kinase A; PLA2: phospholipase A2; PRISMA: Preferred Reporting Items for Systematic Reviews and Meta-Analyses; PUFA: poly-unsaturated fatty acid; ROS: reactive oxygen species; SOD: superoxide dismutase; TAC: total antioxidant capacity; TBA: thiobarbituric acid

## Introduction

Reactive oxygen species (ROS), the unavoidable by-products obtained from oxygen metabolism, are toxic metabolites that can also exert beneficial effects through regulating vital cell signalling cascades [1]. Intracellular ROS concentrations are determined by the balance between the rates of ROS production and their rates of clearance by various antioxidant defence mechanisms. At normal physiological levels, ROS regulate intracellular signalling cascades, thus mediating essential physiological mechanisms such as sperm maturation, hyperactivation, capacitation, acrosome reaction, as well as fertilisation [1,2]. However, when the ROS concentration exceeds the physiological limit, problems occur. Antioxidants are capable of negating such harms; however, when ROS generation overwhelms the antioxidants’ threshold of ROS clearance, or when antioxidant production is diminished, a state of oxidative stress (OS) ensues [3]. This imbalance in the redox potential carries significant negative effects on various cellular components such as: carbohydrates, nucleic acids, proteins, and lipids [4].

The spermatozoa are particularly susceptible to OS owing to their inadequate cell repair systems, as well as insufficient antioxidant defences due to very little cytoplasmic content. They are susceptible to lipid peroxidation (LPO) due to the high content of poly-unsaturated fatty acids (PUFAs) in their plasma membrane, resulting in disruption of membrane permeability, and thus efflux of ATP, impairing flagellar movement [5,6]. A number of studies have confirmed the detrimental impact of OS on semen parameters and fertility potential. Sperm viability, motility, and fertilisation potential are disrupted by OS in the reproductive tissues, evidenced by the presence of significantly higher levels of ROS in the semen of infertile men when compared to fertile controls [7].

Measurement of OS during the evaluation of infertile men is being increasingly practiced, as current evidence has established its utility in various clinical presentations [8]. OS has been detected in patients with unexplained [9] and idiopathic male infertility [8]. Furthermore, conditions such as varicocoele [10,11], infection, inflammation [12], and spinal cord injury [13], have been associated with OS-induced male infertility, underscoring the importance of OS testing in these clinical scenarios. The assessment of seminal OS levels over time could also help in monitoring the responses to antioxidant therapies and define effective doses and durations of treatment.

More than 30 different assays for measuring seminal OS have been described in the literature. These are either direct tests (measuring the degree of oxidation within the sperm cell membrane) or indirect (estimating the detrimental effects of OS, such as DNA damage or LPO levels) [14]. Despite the growing body of evidence, OS testing is not routinely indicated for the evaluation of infertile men. Reasons are principally related to test availability, complexity, cost-effectiveness, and, more importantly, lack of a universally accepted test protocol.

Our present review aims to provide an understanding of the methods of generation of ROS in the male reproductive system, along with their beneficial and deleterious effects on male reproductive functions. It also explores the tools available for the assessment of OS, vital to understand for the clinical corrections of ROS-induced male infertility.

## Methods and materials

**Figure 1.**
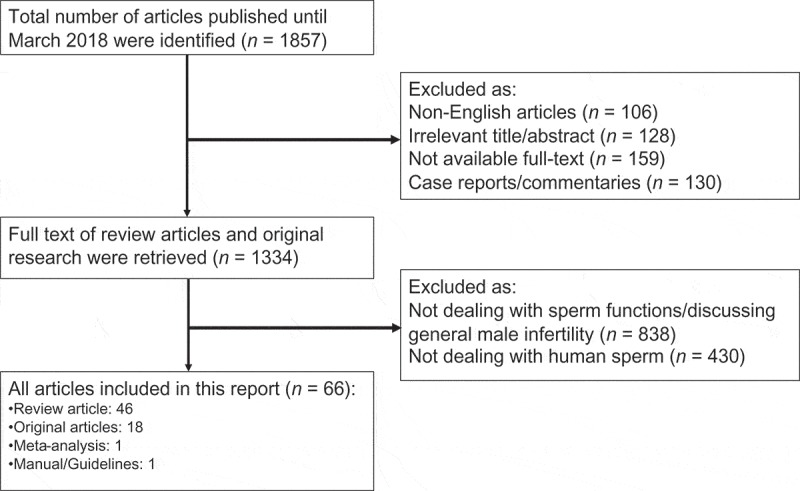
Flow chart of study selection according to PRISMA checklist.

An extensive search of studies published until March 2018 was carried out in the databases of PubMed, Medline, EMBASE, and the Cochrane Library, as per the Preferred Reporting Items for Systematic Reviews and Meta-Analyses (PRISMA) guidelines (Figure 1) [15]. The search was conducted with various combinations of the following keywords: ‘reactive oxygen species’, ‘oxidative stress’, ‘male infertility’, ‘spermatozoa’, ‘oxidative biomarkers’, and ‘sperm functions’. Articles were evaluated based on their title or abstract, and relevant original research studies and review articles were included in this systematic review. Emphasis was placed on studies addressing the following topics: generation of ROS, physiological role of ROS on sperm function, pathological role of ROS in sperm function, evaluation of ROS, and management of OS. Articles were excluded if they were not written in English. We excluded commentaries and case reports.

## Results

In all, 1857 articles were identified. After the first screening, 523 articles were excluded as they were not published in the English language; there was irrelevancy of the title or unavailability of the full text. A total of 1334 articles (reviews and original research) were retrieved, of which 1268 articles were excluded after review, as they did not discuss sperm function in relation to OS, or the general effects of OS on male infertility (Figure 1). The remaining 66 articles were selected as eligible for the present review: 46 of them were systematic and literature reviews, there was one meta-analysis, 18 original articles, and one guideline article.

### ROS generation in male reproductive tissues

**Figure 2.**
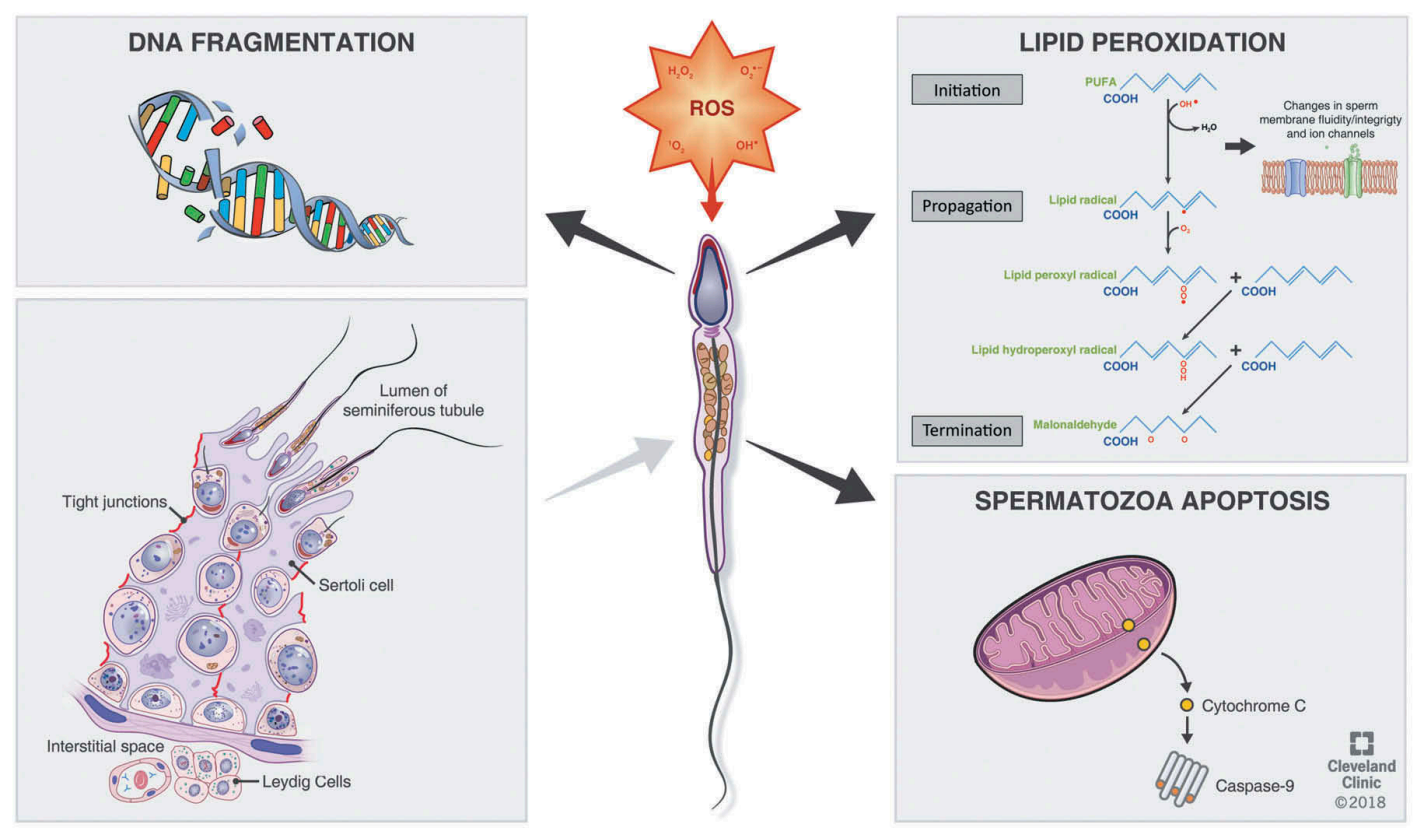
Generation of ROS in spermatozoa.

Spermatozoa can generate ROS mostly via two methods: (a) At the sperm plasma membrane, ROS may be produced by the nicotinamide adenine dinucleotide phosphate (NADPH) oxidase system and/or (b) at the mitochondrial level, ROS are generated via the NAD-dependent redox reaction, which is the most predominant mechanism. Spermatozoa are mitochondria rich cells, owing to their constant requirement of energy for their motility [16]. An increase in the number of dysfunctional spermatozoa in semen significantly induces higher ROS production, affecting its mitochondrial function and motility. The prime ROS in human spermatozoa is superoxide (O_2_^–^), which reacts with itself through dismutation reactions to yield hydrogen peroxide (H_2_O_2_). If transition metals, such as iron and copper, are present H_2_O_2_ and O_2_^–^can generate the most destructive and extremely reactive hydroxyl radical (OH^–^) via the Haber–Weiss reaction (Figure 2), which can initiate a LPO cascade disrupting membrane fluidity and impairing sperm function [17,18].

### Endogenous sources of ROS in seminal plasma

#### Leucocytes

Peroxidase-positive leucocytes [polymorphonuclear leucocytes (~50–60%) and macrophages (∼20–30%)] originate from the male prostate and seminal vesicles. Infectious or inflammatory responses can trigger these cells, which in turn can produce 100-times more ROS than normal as part of the defence mechanisms and also elevate NADPH production through the hexose-monophosphate shunt [4]. Also, the elevation of pro-inflammatory mediators and reduction of antioxidants seen in inflammatory reactions can induce a respiratory burst resulting in OS [19]. Leucocytospermia is a sperm disrupting disorder characterised by the presence of >1 million peroxidase-positive leucocytes/mL of semen [20].

#### Immature spermatozoa

Under normal circumstances, the cytoplasm gets extruded from the developing spermatozoa to prepare itself for fertilisation. However, an arrest to spermatogenesis may result in retention of excess cytoplasm around the midpiece of the damaged spermatozoon (excess residual cytoplasm). Excess residual cytoplasm is capable of activating the NADPH system via the hexose-monophosphate shunt, which is a source of electrons for ROS production and potentially OS [21].

#### Varicocoele

Varicocoele, characterised by an abnormal venous dilatation in the pampiniform plexus around the spermatic cord, is detected in ~40% of male partners of all infertile couples and is thought to be the leading cause of male factor infertility [22]. Many mechanisms have been postulated in the pathophysiology of varicocoele. Testicular hyperthermia and hypoxia are the most commonly accepted theories resulting in OS-induced testicular dysfunction [22,23]. One meta-analysis confirmed the presence of significantly higher OS parameters, such as ROS and LPO, in semen samples from infertile men with varicocoele compared with normal fertile donors [22,24]. The seminal ROS levels have been reported to be directly associated with the grade of varicocoele [25].

### Exogenous sources of ROS

Radiation from mobile phones can induce ROS in human semen, impairing semen quality and inducing sperm DNA damage, thus affecting sperm count, motility, and vitality [26,27]. Radiofrequency electromagnetic waves can impair the intracellular electron flow along internal membranes due to numerous cytosolic charged molecules, thus disrupting normal functioning of the germ cells [13,28,29].

Toxins from domestic or industrial products may impact the body and induce ROS production in the testes, impairing sperm structure and function. Phthalates (in plastic objects), as well as metals such as cadmium, chromium, lead, manganese, and mercury, have been found to impair spermatogenesis, sperm quality and count [28,30].

Smoking causes an imbalance between ROS and antioxidants in the semen of smokers. Smoking may increase seminal leucocyte concentrations by 48% and seminal ROS levels by 107%, decrease seminal plasma antioxidants, and increase 8-hydroxy-2‘-deoxyguanosine (8-OHdG) concentrations (a biomarker of oxidative damage) [28]. Furthermore, smoking induces increased blood and semen cadmium and lead concentrations, which may exaggerate ROS production and impair sperm motility [28]. Increased sperm DNA damage and apoptosis are commonly identified in smokers and have detrimental effects on male fertility [31].

Alcohol is a promoter of ROS generation and also affects the antioxidant defence mechanism. Acetaldehyde, a by-product of ethanol metabolism, can produce ROS by interacting with proteins and lipids, thus, damaging cellular components and decreasing the percentage of normal spermatozoa [32].

### Effects of ROS on different sperm functions

#### Physiological functions

**Figure 3.**
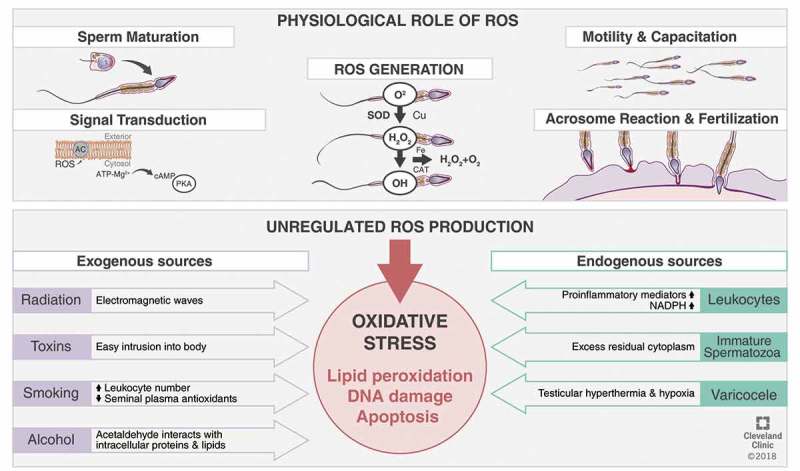
OS in male reproduction.

As mentioned previously, higher concentrations of ROS can have detrimental effects on semen quality, ultimately resulting in infertility. Nonetheless, low and regulated concentrations of ROS play vital physiological roles in male reproduction, such as sperm capacitation, hyperactivation, acrosome reaction, as well as sperm–oocyte fusion (Figure 3) [1,33–38].

#### Maturation

Spermatozoal maturation occurs in the epididymis and is characterised by alterations of the cell membrane, rearrangement of surface proteins, together with nuclear and enzymatic remodelling [1]. This vital step in sperm development is regulated by cellular signal transduction mechanisms that are influenced by ROS levels [36,37]. Chromosomal DNA in the mammalian spermatozoon is densely packed, as its histones are replaced by smaller sized protamines. Inter- and intra-molecular disulphide bonds are established between cysteine residues of protamines to convey chromatin stability. ROS may assist disulphide bond formation, ensuring chromatin stability and protecting DNA from damage. Peroxides may also aid proper formation of the ‘mitochondrial capsule’, which is composed of a protein network rich in disulphide bonds, to secure mitochondria from proteolytic degradation [1,39].

#### ROS as signal transducers

ROS, owing to their small size, ubiquitous nature and short half-life, aid sperm function at different physiological phases such as maturation, activation, capacitation and acrosome reaction [36]. The underlying mechanism of action may be via the redox regulation of cysteine residues. The redox states of the thiol groups determine enzymatic activity. ROS activates adenylate cyclase inducing intracellular cyclic adenosine monophosphate (cAMP) production, which in turn activates protein kinase A (PKA) molecules. PKA mediates the activation of various downstream pathways according to the spermatozoa maturational state [1].

#### Motility and hyperactivation

Hyperactivation is a particular state of sperm motility characterised by high amplitude, increased and asymmetric flagellar movement, elevated side-to-side sperm head displacement, along with non-linear motility [40]. It is considered to be part of capacitation and is required for successful sperm penetration of the zona pellucida and fertilisation. ROS has positive impacts on the hyperactivation processes in spermatozoa [37]. The initiation process of capacitation and hyperactivation is induced by the influx of Ca^2+^ and HCO_3_^–^, probably by the inactivation of an ATP-dependent Ca^2+^-regulatory channel (plasma membrane Ca^2+^-ATPase, PMCA) and alkalisation of the cytosol. Calcium ions and ROS, specifically O_2_^–^, lead to the activation of adenylate cyclase, generating cAMP. cAMP via PKA activation triggers NADPH oxidase and thereby stimulates greater ROS generation. PKA also phosphorylates serine and tyrosine residues, which can also activate protein tyrosine kinase (PTK). Consequently, PTK triggers phosphorylation of tyrosine residues in the fibrous sheath around the axoneme and the cytoskeleton of the sperm flagellum. ROS, especially H_2_O_2_, elevate tyrosine phosphorylation by inducing PTK and inhibiting phosphotyrosine phosphatase (PTPase), which leads to de-phosphorylation of tyrosine residues. The final step in the process of hyperactivation is presumably increased tyrosine phosphorylation [1]. O_2_^–^ has been observed to be the major ROS contributor to this ameliorating effect [28].

#### Capacitation

The ultimate functional process in spermatozoal maturation needed for making the sperm competent to fertilise an ovum is capacitation. The established molecular pathway by which ROS facilitates capacitation is by triggering intracellular cAMP levels inducing downstream PKA, which in turn phosphorylates MEK (extracellular signal regulated kinase)-like proteins, threonine-glutamate-tyrosine, and fibrous sheath proteins [41,42]. These signalling cascades bring about final capacitation of the sperm rendering it totally prepared for the acrosome reaction [28,41].

#### Acrosome reaction

To ensure fertilisation, the hyperactivated spermatozoon must pass across the cumulus oophorous, bind to the zona pellucida of the oocyte and create a pore in its extracellular matrix via exocytotic release of proteolytic enzymes [39]. These acrosome reactions are mediated through phosphorylation of tyrosine proteins, Ca^2+^ influx resulting in an intracellular rise in cAMP and PKA, thereby enabling the spermatozoon to penetrate and fuse with oocyte. ROS has been observed to facilitate actions on the zona pellucida of the spermatozoon by various means including phosphorylation of three relevant plasma membrane proteins [1,36].

#### Sperm–oocyte fusion

ROS appear to increase membrane fluidity required for successful sperm–oocyte fusion after aiding the biochemical cascades of spermatozoa capacitation and acrosome reactions. Throughout capacitation, ROS prevents deactivation of phospholipase A2 (PLA2) by inhibiting protein tyrosine phosphatase activity, so that PLA2 can cleave the secondary fatty acid from the membrane phospholipid triglycerols to increase fluidity of the membrane [43].

### Pathological functions

When the highly reactive ROS overpower the antioxidant defence systems and disturbs the homeostatic balance between ROS generation and antioxidants activities [38], pathological defects arise in vital biomolecules such as proteins, nucleic acids, lipids, and sugars (Figure 2) [32,35,37,44].

#### Lipid peroxidation (LPO)

The sperm cell is characterised by having high levels of lipids in its plasma membrane, mostly in the form of PUFAs having unconjugated double bonds between their methylene groups. The double bond near to the methylene group reduces the strength of the methyl carbon–hydrogen bond, rendering hydrogen exceedingly susceptible to oxidative damage. As the intracellular levels of ROS rise uncontrollably, they initiate a cascade of reactions ultimately resulting in LPO [23,44,45], in which almost 60% of the membrane fatty acids are lost, diminishing its fluidity, enhancing non-specific permeability to ions, and also inhibiting the actions of membrane receptors and enzymes. LPO is thus an autocatalytic self-propagating chemical reaction leading to abnormal fertilisation, and the mechanism of this oxidative damage may proliferate through three major steps, namely: initiation, propagation, and termination [28,45,46].

Initiation includes hydrogen atoms abstraction from the carbon–carbon double bonds thus propelling free radicals, which in turn generate lipid radicals, and the later react with oxygen forming the peroxyl radicals [45]. These peroxyl radicals may again abstract hydrogen atoms from the lipids, especially when metals such as copper and iron are present, progressing the chain of autocatalytic reaction. The propagation stage of oxidative damage continues with the formed radicals reacting with successive lipids, generating cytotoxic aldehydes owing to degradation of hydroperoxide [18,41,45]. The formation of peroxyl and alkyl radicals proceed in a cyclical manner in this propagation step until a stable end product is formed, which is malondialdehyde (MDA) and the reaction chain reaches its termination [45]. Thus, MDA is an essential biochemical marker for analysing and monitoring the level of peroxidative damage affecting the spermatozoa. Another product of LPO is 4-hydroxynonenal, which is hydrophilic and can lead to severe spermatozoa dysfunction at both the proteomic as well as genomic levels [28].

#### DNA damage

Several deleterious effects of ROS on sperm nuclear DNA are evident owing to increased DNA fragmentation, chromatin cross-linking, base-pair (bp) modifications, and chromosomal microdeletions [28,45,47]. ROS are also responsible for reduced sperm motility by inhibiting energy generation, via LPO and importantly mitochondrial DNA (mtDNA) mutations. Damage to at least one of the 13 genes coding for the electron transport chain transporter system in the mitochondria will reduce ATP production and induce intracellular ROS production [28]. ROS may reduce sperm motility also by oxidation of a thiol group in glyceraldehyde-3-phosphate dehydrogenase (GAPDH), which is a glycolytic enzyme, or deletion of adenine and pyridine nucleotides by LPO [28,47].

#### Apoptosis

ROS are capable of disrupting the inner and outer mitochondrial membranes releasing cytochrome C. This cytochrome C in turn activates the apoptotic caspases [28,42]. This mechanism of induction of apoptosis in the spermatozoa by ROS is evident in infertile men, as high levels of cytochrome C have been found in the seminal plasma of infertile men, which is an indicator of severe mitochondrial damage [27,28].

### Evaluation of seminal OS

ROS-mediated damage to sperm is evidently a major contributing pathology in 30–80% of men with unexplained infertility [48]. Therefore, analysis of elevated ROS levels in infertile men is quite reasonable. Factors, such as inconvenience of ROS screening, its high cost, and lack of overall accepted efficient analysis method, hinder consideration of ROS measurement as an integral part of male infertility assessments, despite its immense importance. At present, >30 different assays have been described in the literature for measuring ROS and OS in the semen of infertile men (Table 1) [28].

**Table 1.** OS testing methods.

Testing method	Explanation
Routine semen analysis	Parameters of routine semen analysis can suggest the presence of OS, these include: Asthenozoospermia Abnormal morphology and retained cytoplasmic droplets Poor sperm membrane integrity Hyperviscosity Leucocytospermia
ROS measurement by chemiluminescence	The procedure involves a luminometer and a chemiluminescent probe such as luminal (5-amino-2,3,-dihydro-1,4-phthalazinedione). The free radicals contained in the semen sample, produce a light signal reacting with luminal, which is converted by the luminometer to an electric signal (photon).
TAC measurement by chemiluminescence	Luminol is also used for the measurement of the TAC within the seminal plasma, which is quantified against a vitamin E analogue ‘Trolox’ (a water-soluble tocopherol analogue).
LPO markers	The most commonly measured decay product is MDA, which is mediated by TBA assay where MDA combines with TBA producing a 1:2 adduct, a coloured substance measured by fluorometry or spectrophotometry
Seminal ORP	Also known as the redox potential, this is a measure of the potential for electrons to move from one chemical species to another. The MiOXSYS measures the balance between oxidants and reductants in seminal fluid.

MDA, malondialdehyde; MiOXSYS, Male Infertility Oxidative System; ORP, oxidation–reduction potential; OS, oxidative stress; ROS, reactive oxygen species; TAC, total antioxidant capacity; TBA, thiobarbituric acid.

#### Routine semen analysis

The routine analysis of semen parameters (sperm count, morphology and motility) allows clinicians to make an almost perfect diagnosis of OS, where asthenozoospermia is perhaps the best marker for OS [49,50]. The hyperviscosity of seminal plasma marks a rise in seminal plasma MDA and a decrease in seminal plasma antioxidant status [49]. Moreover, *Ureaplasmaurealyticum* infection in the semen is also associated with high viscosity of seminal plasma and high ROS production [49]. The detection of a considerable number of round cells may signify the presence of leucocytospermia, which is a well-known source of exaggerated ROS production, as stated previously. However, to ensure that the round cells are not immature spermatozoa, ancillary tests such as the peroxidase test, seminal elastase measurement or cluster of differentiation 45 (CD45, a transmembrane glycoprotein expressed on the cell surface) antibody staining, should be done. Disrupted sperm morphology and cytoplasmic droplets are prime features of anomalous spermatozoa leading to uncontrolled production of ROS. Lastly, poor sperm membrane integrity, which may be assessed by the hypo-osmotic swelling test (HOST), has been linked to the presence of OS [28].

#### ROS by chemiluminescence

Seminal ROS measurement is mostly assessed by the chemiluminescence assay. The procedure involves a luminometer and a chemiluminescent probe such as luminal (5-amino-2,3-dihydro-1,4-phthalazinedione; Sigma-Aldrich, St. Louis, MO, USA). Aliquots of liquefied semen are centrifuged at 300 ***g*** for 7 min, followed by freezing the aliquoted seminal plasma at −20°C for measurement of the total antioxidant levels. The pellet is then washed using PBS (pH 7.4) and 400-μL aliquots of 2 × 10^6^ sperm/mL are re-suspended in the washing medium and used for the assessment of basal ROS levels. The negative control contains 10 mL of 5-mM luminol in 400 mL of PBS. Luminol (5-mM stock in dimethyl sulphoxide) is added to the mixture to serve as a probe and the test tubes are loaded in the luminometer for 15 min to measure the level of ROS. Luminol measures both extracellular and intracellular ROS. The free radicals contained in the semen sample, produce a light signal reacting with luminal, which is converted by the luminometer to an electric signal (photon). The measurement of the number of free radicals generated is done as relative light units/s/10^6^ sperm. The range of normal ROS levels in washed sperm suspensions is 0.10–1.03 × 10^6^counted photons per minute per 20 × 10^6^ sperm [50].

#### Total antioxidant capacity (TAC)

Luminol is also used for the measurement of the TAC within the seminal plasma, which is quantified against a vitamin E analogue ‘Trolox’ (a water-soluble tocopherol analogue). The results are articulated as a ROS-TAC score, indicating the combined antioxidant activities evoked by all the constituents, including vitamins, lipids and proteins [50].

#### LPO markers

Lipid peroxides accumulation in the spermatozoa produce a variety of decay end-products such as MDA, 2‑propenal (acrolein), hydroxynonenal, and isoprostanes, which can be measured as indicators of OS [45]. MDA measurement, the most commonly used method, is mediated by the thiobarbituric acid (TBA) assay, where MDA combines with TBA producing a 1:2 adduct, a coloured substance measured by fluorometry or spectrophotometry [28,50].

#### Seminal oxidation–reduction potential (ORP)

The ORP, also known as the redox potential, is a measure of the potential for electrons to move from one chemical species to another [51,52]. ORP is a measure of this relationship between oxidants and antioxidants, providing a comprehensive measure of OS. Recently, a novel technology based on a galvanostatic measure of electrons has been developed, and it has been used to assess changes in OS in trauma patients and as a function of extreme exercise [53,54]. ORP in the semen has been easily and comprehensively measured using the Male Infertility Oxidative System (MiOXSYS®, Aytu BioScience Inc., Englewood, CO, USA) that enables wider application of OS analysis in clinical and research settings [55]. ORP results provided by the MiOXSYS are standardised, reliable and reproducible compared to previously used ROS assays [55,56].

## Implications on conventional semen parameters and advanced sperm function tests

Several studies have confirmed the presence of significant correlations between altered semen parameters and exaggerated levels of ROS, indicating that OS has a negative impact on male fertility. Homa et al. [57] compared seminal ROS levels in three study groups: Group 1, normal semen parameters without leucocytospermia; Group 2, abnormal semen parameters without leucocytospermia; and Group 3, any semen parameters with leucocytospermia. Expectedly, the highest ROS levels were detected in Group 3 patients; however, Group 2 had significantly higher ROS levels than Group 1 patients, indicating an inverse association between ROS and normal semen parameters. This finding has also been reproduced by Agarwal et al. [58], who examined semen parameters and ROS levels in the ejaculates of infertile men, as well as men with confirmed fertility. The authors reported significantly higher levels of ROS amongst infertile men compared with fertile controls. They detected significant positive correlations between ROS levels and sperm concentration and motility amongst all men, as well as in infertile men and fertile controls. Another study examined seminal MDA, nitric oxide (NO), zinc and TAC levels in infertile and fertile men. Seminal levels of MDA and NO were significantly higher in infertile than fertile men. Whilst seminal levels of zinc and TAC were significantly lower in infertile than fertile men [59]. Sperm concentration, motility and morphology showed significant negative correlations with MDA and NO and significant positive correlations with zinc and TAC.

Venkatesh et al. [60] investigated the correlation between various sperm morphological defects with seminal OS in infertile and fertile men. The authors found a median ROS level that was up to 124-times higher in the infertile men compared with the fertile men. Amongst the various morphological defects, the percentage of sperm with cytoplasmic droplets was found to be significantly higher in infertile men compared with fertile men, which may be a consequence of the elevated ROS levels in their semen samples. A negative effect for OS on acrosomal structure was confirmed in another study, after observing a significant positive correlation between acrosomal anomalies and MDA values [61].

Current evidence generally recognises seminal OS as an important cause of sperm DNA fragmentation, suggesting that testing for sperm DNA fragmentation could be valuable in the evaluation of male infertility [62,63]. Iommiello et al. [64] reported a significant positive correlation between OS [measured with oxiSperm® (Halotech DNA, SL, Madrid, Spain)] and sperm DNA fragmentation index [measured with sperm chromatin structure assay(SCSA®; SCSA Diagnostics, Volga, SD, USA)]. Another study reported a significantly higher percentage of spermatozoa with fragmented DNA in infertile men compared with fertile men, and confirmed the presence of significant positive correlations between sperm DNA fragmentation and seminal MDA levels [65]. In a recent study, we assessed the influence of seminal ORP and sperm DNA fragmentation (measured with sperm chromatin dispersion) on sperm morphological anomalies amongst infertile and fertile men. In our study, the ORP and sperm DNA fragmentation showed significant positive and significant negative correlations with sperm head defects and normal morphology in infertile men, respectively. Moreover, ORP and sperm DNA fragmentation were inversely associated with the level of normal sperm morphology (both *P *< 0.001) [66].

## Prevention and management of male reproductive OS

The first step in the management of OS should be to verify the underlying cause(s) of the imbalance between the ROS load and antioxidant level in order to deliver effective treatment strategies. Some important management factors are discussed below.

### Lifestyle modifications

Increased professional and personal stresses, mostly attributable to a developed society, lead to bad habits such as: substance abuse, smoking, and an unbalanced diet, which are all recognised as potential causes for OS. Therefore, minimising such behaviours should aid in OS alleviation [28,67]. In addition, exposure to pollution, heat, toxins, heavy metals etc., contribute largely to the development of OS. Apart from these, any other activities raising the scrotum’s temperature such as, saunas, hot baths, long period of driving, and long sedentary office hours, should be monitored. There should be adequate aeration and protection at work places to limit exposure to any noxious chemicals or vapours that may potentiate OS [23,48,68].

### Antioxidants

Antioxidants eliminate ROS or reduce their formation to halt the oxidative chain reaction. Preventive antioxidants (metal chelators or binding proteins), such as lactoferrin and transferrin, inhibit the formation of ROS [69]; whereas scavenging antioxidants, such as vitamins C and E, eliminate ROS [70]. Antioxidants can also be enzymatic and non-enzymatic. Enzymatic antioxidants include natural antioxidants such as: glutathione reductase (GSH), superoxide dismutase (SOD), and catalase; whilst, some important non-enzymatic antioxidants include vitamins C, E, and B; carnitines; cysteines; carotenoids; pentoxifylline; taurine; metals; hypotaurine; and albumin. The non-enzymatic antioxidants can be acquired from foods containing the supplements [28,71,72].

Several studies have investigated the effect of antioxidant supplementation on semen parameters and measures of OS in seminal plasma [73–77]. One literature review by Gharagozloo and Aitken [78] identified 20 trials highlighting the effects of antioxidant treatment on measures of OS in human spermatozoa. The authors reported a significant reduction in OS or DNA damage after treatment with antioxidants in 19 out of the 20 studies selected. Moreover, an improvement in sperm motility, particularly in asthenospermic men was significantly observed [78]. Another systematic review by Majzoub and Agarwal [79] identified the antioxidants vitamin E, vitamin C, N-acetyl-cysteine, selenium, and zinc, to be particularly advantageous for the treatment of OS-related male infertility.

### Surgery

Varicocoele is marked by abnormal elongation and dilatation of the pampiniform plexus of veins surrounding the spermatic cord. Corrective surgery occludes these dilated veins in subfertile males or male patients with testicular pain. This technique has been reported to reduce seminal ROS levels protecting the sperm from oxidative damage [48]. Surgical repair also ameliorates other major biomarkers of infertility, including sperm parameters as well as successful pregnancy rates [11,28]. Recent evidence has also suggested surgical repair of varicocoele for the treatment of hypogonadism in infertile men [80]. Mostafa et al. [81] measured MDA, H_2_O_2_ and NO, as well as six antioxidants [SOD, catalase (CAT), glutathione peroxidase, vitamin C, vitamin E, albumin] 1 day before varicocoelectomy, and at 3 months and 6 months after surgery. There was a statistically significant reduction in all measured ROS and seminal levels of four out of the six antioxidants (SOD, CAT, glutathione peroxidase, and vitamin C) significantly increased at 3-months postoperatively compared with the preoperative values, with a further significant reduction during the subsequent 3 months. The authors concluded that varicocoelectomy reduces ROS levels and increases antioxidant activity of seminal plasma from infertile men with varicocoele. Chen et al. [82] assessed the effect of varicocoelectomy on 4977 bp deletion of mtDNA and 8-OHdG content in spermatozoa DNA (both measures of OS-induced sperm DNA damage) in 30 young subfertile men. After varicocoelectomy, the authors reported a statistically significant reduction in the above mentioned measures, further highlighting the beneficial effect of surgery on OS-induced male infertility.

## Conclusion

OS results from disturbances in the intricate balance between ROS generation and elimination. Whilst physiological levels of ROS are vital for optimal sperm function; when present in exaggerated levels, ROS may have detrimental effects on sperm quality and function, and ultimately result in infertility. Several markers and measurement methods for OS have been described such as: chemiluminescence measurement of ROS and TAC, LPO markers, and ORP, and their assessment provides valuable information during the evaluation of infertile men. Effective prevention and treatment of OS via lifestyle changes, antioxidant supplementation and varicocoelectomy, when indicated, are essential to improve the reproductive potential of infertile men.
